# Marital Cohesion and Perceived Stress in Parents of Children with Special Educational Needs: A Study of the Impact on Couple Dynamics

**DOI:** 10.3390/children12040436

**Published:** 2025-03-29

**Authors:** Sandra Figueiredo, Raquel Pereira

**Affiliations:** Foundation for Science and Technology, Universidade Autónoma de Lisboa, Palácio Dos Condes Do Redondo, R. de Santa Marta 56, 1169-023 Lisbon, Portugal; raquelpereira1310@gmail.com

**Keywords:** special educational needs, marital cohesion, flexibility, perceived stress

## Abstract

Background/Objectives: The presence of a child with special educational needs (SENs) necessitates that both the child and their family engage in adaptive processes and develop resilience in response to the developmental challenges that arise following an SENs diagnosis. Furthermore, it is critical to examine the impact of such a diagnosis on parental marital cohesion. Despite the recognized importance of this issue, the effects of SENs diagnoses on marital cohesion and flexibility, and the perceived stress experienced by parents, remain underexplored in the extant literature. Methods: Forty-seven participants (23 parents of children diagnosed with SENs and 24 parents of typically developing children, all aged between 32 and 60 years) completed this study. The instruments used were the Family Cohesion and Flexibility Evaluation Scale (couple version) and the Perceived Stress Scale. Results: No direct relationship was found between the SENs diagnosis and marital cohesion or flexibility. Furthermore, the perceived stress did not mediate the association between these variables. Nonetheless, both groups exhibited high levels of perceived stress. Conclusions: These results underscore the necessity for further investigation into the role of emotional support and coping strategies in alleviating the perceived stress among parents and caregivers of children with SENs. A deeper understanding of these factors is essential for the development of interventions aimed at promoting parental well-being and enhancing the quality of caregiving.

## 1. Introduction

Parenting a child with special educational needs (SENs) constitutes a significant challenge, as it introduces fear and uncertainty and requires profound adjustments within the family system, thereby impacting marital relationships and contributing to heightened levels of perceived stress among parents [1,2,3] (Araújo & Duarte, 2012; McConnell et al., 2015; Di Renzo et al., 2021). SENs encompass a broad spectrum of developmental challenges that affect a child’s functioning in both educational and developmental contexts [4,5,6,7,8]. Both temporary and permanent SENs are carefully considered throughout the learning process, along with the necessary adaptations to assure the inclusion of all children. These adaptations vary solely based on the specific modifications required to meet the children’s needs. Temporary SENs typically require targeted, short-term adaptations to the curriculum, demanding an inclusive instructional approach over a defined period [7,8]. Permanent SENs necessitate extensive and sustained modifications to the curriculum, which are intended to persist throughout much or all of the child’s academic trajectory [9,10].

Previous studies, such as that by Tsibidaki and Tsamparli [11], have explored differences in cohesion and flexibility between families with children with SENs and families with typically developing children, concluding that there are no statistically significant differences between the two groups. In contrast, an increasing number of studies have demonstrated that families with children with SENs exhibit higher levels of cohesion and flexibility compared to families with children without such diagnoses [12,13]. However, a comprehensive view on the relationship between SENs and the cohesion and flexibility of parents and caregivers, and their impact, are not yet well understood. On the one hand, most studies predominantly focus on autism spectrum disorder (ASD), often neglecting other conditions that similarly affect family dynamics, such as anxiety disorders, cerebral palsy, and learning disabilities. This study aims to address this gap by adopting a broader perspective on SENs, considering a wider range of diagnoses. It evaluated the impact of a SENs diagnosis on marital cohesion and flexibility, comparing parents of children with SENs to those of typically developing children, in Portugal. On the other hand, this research investigated the mediating role of perceived stress in the relationship between these variables—an area that has been largely overlooked in previous studies—providing a more comprehensive understanding of the psychological mechanisms involved.

Regarding stress perceived by parents, most studies show that parents of children with SENs experience higher levels of stress, which can negatively impact their mental health and family well-being [14,15]. In addition, higher levels of stress are observed among women, as the task distribution remains, in most cases, unequal, with mothers often assuming the primary caregiving role [16,17]. Further samples and cross-cultural contexts should be considered to understand the prevalence of the association of the stress of parents with existing SENs in children in their household [18,19]. A lack of evidence is visible in this context.

Therefore, in this context, it is important to highlight the persistent inequality in Portugal regarding the distribution of household tasks between men and women, which contributes to higher stress levels among women [17]. This imbalance is particularly relevant in the context of special educational needs (SEN), where mothers often bear the primary responsibility for childcare, household management, and maintaining employment. The strong societal expectation that women should be the main caregivers further exacerbates their perceived stress, impacting both their mental health and the quality of their marital relationships.

Furthermore, the availability of support services and early intervention for children with SENs remains uneven, particularly in rural areas, where access to such services is more limited. These constraints make the Portuguese context particularly complex, underscoring the need to investigate how stress is experienced differently across countries, considering the cultural and policy-specific factors that shape the experiences of the families of children with SENs.

Exploring the mental health of parents and caregivers of children with SENs is vital, as their well-being plays a central role in their capacity to manage the emotional demands associated with the diagnosis, which in turn influences the quality of caregiving provided to the child. The present study aims to examine the impact of SENs on the marital cohesion, flexibility, and stress perceived by parents, by comparing couples with and without children diagnosed with SENs, specifically in Portugal

## 2. Method

- **Hypotheses**

1. Parenting children with SENs is associated with lower marital cohesion;
2. Parents of children with SENs exhibit greater flexibility;
3. Higher perceived stress among parents of children with SENs compared to parents of typically developing children;
4. Perceived stress mediates the relationship between the child’s diagnosis (SENs) and the parents’ cohesion and flexibility.

### 2.1. Participants

The inclusion criteria for participating in this study were: (1) individuals aged 18 years or older (2) who are married or cohabiting and (3) who have one or more children from their current relationship, aged between 5 and 18 years. The age range of 5–18 years was based on the developmental and educational challenges typically associated with this period, which aligns with the formal identification of SENs. Children under 5 were excluded, as diagnoses are often not yet established at this stage, and early development can present complex challenges that may contribute to heightened perceived stress in parents. This focus ensured a more homogeneous sample in terms of the developmental stage and parental demands. The sample consisted of 47 Portuguese participants, aged between 32 and 60 years (M = 44.21; DP = 5.714), with 31 female participants (66%) and 16 male participants (34%). Twenty-three individuals were children with SENs (48.9%), of whom 15 were female and 8 were male, and 24 individuals were children without this diagnosis (51.1%), of whom 16 were female and 8 were male. Regarding the school, it is an inclusive institution that accommodates both children with and without special educational needs. It has approximately 400 students, distributed across 27 classes ranging from nursery to the 9th grade.

- **Families with children with SENs**

In the group of participants with children with SENs, the majority had obtained a bachelor’s degree (47.8%), with educational levels ranging from basic education (4.3%) to a master’s degree (13%). Regarding marital status, 56.5% were married, and 43.5% were cohabiting. The number of children ranged from 1 to 3, with the majority having just one child (52.2%). The average age of the first child was 11.30 years, and was 7.55 for the second child and 6 years for the third child. Diagnoses of SENs included visual impairment (*n* = 7), autism spectrum disorder (*n* = 5), developmental coordination disorder (*n* = 3), cerebral palsy (*n* = 2), obsessive-compulsive disorder (*n* = 2), anxiety disorder (*n* = 2), dysorthography (*n* = 1), and septo-optic dysplasia (*n* = 1).

Within the context of permanent disabilities associated with SENs, this study will focus on the primary diagnoses observed within the sample and their respective implications:

Visual impairment (VD) is associated with losses or reductions in anatomical structure or function in the visual field, encompassing cases of low vision and blindness [20].

Autism spectrum disorder (ASD) is a permanent neurodevelopmental disorder characterized by difficulties in communication and social interaction, along with rigid and repetitive patterns of behavior and interests [21,22].

Developmental coordination disorder (DCD) refers to delayed motor development that impacts daily living activities [23]. Additionally, it is associated with alterations in brain function, affecting the execution of tasks that involve executive function [24,25].

Cerebral palsy (CP) refers to injuries to the central nervous system in the development stage, which affect the child’s motor and cognitive function. It is the main cause of physical disability in childhood [26,27].

Obsessive-compulsive disorder (OCD) is associated with obsessions and/or compulsions that severely disrupt daily routines and well-being [28]. Obsessions refer to intrusive thoughts, while compulsions are repetitive behaviors performed in response to an obsession [23].

Anxiety disorder (AD) is characterized by excessive and persistent worry about future events [29].

Dysorthography represents a specific learning disorder with a deficit in written expression, and is a temporary condition [30].

Septo-optic dysplasia (SOD) is a neurological condition characterized by alterations in midline brain structures, hypoplasia of one or both optic nerves, and variations in hypothalamic-pituitary function [31]. Symptoms of SOD affect cognitive development, endocrine function, sleep, and vision [32].

- **Families with children without SENs**

In the group of individuals with children without SENs, the majority held a bachelor’s degree (54.2%), with educational levels ranging from high school (29.3%) to a PhD (4.2%). In terms of marital status, 62.5% were married, and 37.5% were cohabiting. The number of children in this group varied between 1 and 4, with the majority having two children (54.2%). The average age of the first child was 12.71 years, and was 8.88 for the second child, 5.75 for the third child, and 2 years for the fourth child.

### 2.2. Instruments

The present study collected data through a sociodemographic questionnaire, which included questions on age, gender, educational qualifications, profession, marital status, relationship duration, number of children, their ages, and their diagnoses. In addition, this study employed the couple version of the Family Adaptability and Cohesion Evaluation Scales (FACES III) and the Perceived Stress Scale (PSS 10).

- **Family Adaptability and Cohesion Evaluation Scales**

The Family Adaptability and Cohesion Evaluation Scales (FACES III), developed by Olson [33] and adapted for the Portuguese population by Abreu-Afonso and Leal [34], consist of 20 items designed to assess the dimensions of cohesion and flexibility. Divided into two components, it first evaluates participants’ perceptions of their current relationship, referred to as real cohesion and real flexibility, and then captures their idealized vision of the relationship, known as ideal cohesion and ideal flexibility. Its primary objective is to measure marital functioning through the perception and idealization of cohesion and flexibility. Higher or lower scores indicate greater dysfunctionality.

The cut-off points were provided by the authors of the validated Portuguese scale, albeit with discretion regarding their publication. Based on the classification system, scores on the cohesion dimension range from 10 to 50, with lower values (10–34) indicating a disengaged or separated family system, while higher scores (42–50) reflect connected or enmeshed family dynamics. Similarly, in the flexibility dimension, the scores range from 10 to 50, with lower values (10–27) corresponding to a rigid structure and intermediate scores (28–39) indicating structured or flexible adaptability. In contrast, scores between 40 and 50 suggest a chaotic system, characterized by instability and a lack of defined rules. The Cronbach’s alpha coefficient for the original version was 0.68. In this study, the Cronbach’s alpha was 0.936, demonstrating strong internal consistency.

- **Perceived Stress Scale**

The Perceived Stress Scale (PSS 10), developed by Cohen et al. [35] and validated for the Portuguese population by Trigo et al. [36], aims to measure how life events influence participants’ perception of stress. The scale comprises 10 items, rated on a 5-point Likert scale. Scores are classified into three levels: low stress, moderate stress, and high stress, with higher scores indicating greater levels of perceived stress. The cut-off points were established based on the percentile distribution from the Portuguese validation study, albeit with discretion regarding its publication. For example, scores greater than 40 but not exceeding 60—derived from calculated percentiles—indicate the presence of stress (according to the validated Portuguese version). Scores up to 90 are indicative of excessive stress, reflecting burnout and an increased risk of psychosomatic disorders. The Cronbach’s alpha coefficient for the original version was 0.78. In this study, the Cronbach’s alpha was 0.587; however, an examination of the impact of item exclusion on scale reliability did not identify any items whose removal would significantly improve the Cronbach’s alpha coefficient. Thus, all items were retained to preserve the full representativeness of the construct.

### 2.3. Procedures

The first stage of this study involved obtaining authorization from the Chairperson of the School Board, in Lisbon district, Portugal, ensuring the anonymity and confidentiality of the participants. After receiving approval, the school’s teachers and educators shared the link to the online survey with the students’ parents. The survey, hosted on the Google Forms platform, included the informed consent form, which guaranteed confidentiality and emphasized voluntary participation in this study, as well as containing information regarding this study’s objectives, inclusion criteria, and instruments used.

Due to the limited number of participants, it became necessary to expand the survey’s reach by sharing the access link via social media, mostly using researchers’ personal social media networks. This strategy was employed to increase the sample size and ensure a more representative sample of the target population, in order to strengthen the robustness of this study’s results. However, it is important to acknowledge that the use of social media may have introduced a potential sampling bias, as not all individuals have equal access to the internet, and the sample may have overrepresented certain demographic groups.

In order to compare responses between partners (in the same couple), participants were asked to agree on a code with their spouse and write this code in the first section of the questionnaire, allowing for the identification of relationships between respondents. Although some couples complied with this request, it was observed that the vast majority of the provided codes were not repeated by another participant. Consequently, in most cases, only one partner completed the questionnaire, making direct comparisons impossible.

The research project was approved by the Ethics Committee of CUIP guaranteeing compliance with ethical and deontological standards.

### 2.4. Data Analysis

The data were analyzed using IBM SPSS (Statistical Package for the Social Sciences) statistics 29 software.

The analysis began with a descriptive assessment of the sample and variables, exploring absolute and relative frequencies, means, and standard deviations. Next, the internal consistency of the measures was evaluated using Cronbach’s alpha, and the normality of the variables was tested using the Shapiro–Wilk test (*n* < 50). For variables that did not meet the assumptions of normality, such as real and ideal cohesion and ideal flexibility, non-parametric tests were performed, specifically the Mann–Whitney test for group comparisons and Spearman’s correlation for correlation analyses.

On the other hand, normally distributed variables, such as real flexibility and perceived stress, were analyzed using parametric tests, including Student’s *t*-test for mean comparisons and Pearson’s correlation to examine relationships between variables.

Given the small number of participants in each group, statistical analyses were conducted with the highest level of rigor. The combination of parametric and non-parametric tests, according to normality assumptions, ensured that the analyses were appropriate for the data. Nonetheless, the results should be interpreted with caution, and future research with larger samples is recommended to confirm the present findings.

Tests such as Student’s *t*-test, Mann–Whitney, Pearson, and Spearman correlations, mediation using the Hayes (2017) PROCESS model, and simple linear regressions were employed. Results with *p* < 0.05 were considered statistically significant. The interpretation of Pearson and Spearman correlation coefficients followed Cohen’s [35] criteria: weak correlation (<0.30), moderate (>0.30), and strong (>0.50). The Spearman correlations and regression analysis for mediation were the main focus of the present paper.

## 3. Results

### 3.1. Spearman Correlation Between SEN, Real Cohesion, Ideal Cohesion, and Ideal Flexibility

There were no statistically significant differences between the presence of children with SENs and the variables under study (*p* > 0.05): marital and family cohesion, flexibility, and perceived stress. In contrast, significant correlations were found with the other variables, specifically real cohesion, ideal cohesion, and ideal flexibility. These results suggest that the presence of children with SENs may not be directly associated with parents’ perceptions of their marital and family cohesion and flexibility. On the other hand, parents’ expectations regarding their marital and family dynamics may influence their perception and understanding of the reality (see Table 1).

### 3.2. Pearson Correlation Between SEN, Real Flexibility, and Perceived Stress

The results showed that the SENs variable is weakly correlated with the real flexibility variable (r = −0.161), its subscale leadership and control (r = −0.220), and the perceived stress scale (r = −0.136). However, although a negative association was observed between these variables, no statistically significant differences were found (*p* > 0.05). Consequently, the diagnosis of SENs in a child did not appear to have a significant influence or impact on their parents’ real flexibility and perceived stress (see Table 2).

### 3.3. Mediation Analysis

The findings supported that, although the perceived stress by parents appears to negatively influence their marital cohesion (b = −0.6506, *p* = 0.0000, 95% CI [−0.8966, −0.4046], t = 5.3304), no sufficient evidence was found to support the hypothesis that perceived stress mediates the relationship between the presence of children with SENs and the parents’ marital cohesion. While perceived stress appears to have a direct negative impact on marital cohesion, it does not act as the underlying mechanism that links the presence of children with SENs to this particular aspect of marital functioning. This suggests that other factors, such as communication patterns or social support, may better explain how the presence of children with SENs influences marital cohesion (see Table 3).

The results indicated that perceived stress by parents appears to negatively influence marital flexibility (b = −0.4144, *p* = 0.0056, 95% CI [−0.7012, −0.1276], t = −2.9121). However, no sufficient evidence was found to support the hypothesis that perceived stress mediates the relationship between the presence of children with SENs and parents’ flexibility. Despite the significant direct effect of perceived stress on marital flexibility, it did not mediate the influence of children with SENs on this aspect of marital functioning. Therefore, it is important to consider other factors that may be associated, such as coping strategies, social support, and individual characteristics of the parents (see Table 4).

### 3.4. Regression Linear Analysis

Concerning the linear regression analysis, the results revealed that the model was not statistically significant, meaning that it is not possible to assert that the participants’ gender had a significant effect on their perceived marital cohesion. Only 5.5% of the variance in marital cohesion perception was explained by gender. The results indicated that the model was statistically significant, F(1,45) = 7.819, *p* = 0.008, supporting the idea that gender may serve as a predictor of perceived stress. In this context, it is important to note that the females (M = 22.48, SD = 6.787) exhibited significantly higher mean scores than the males (M = 16.75, SD = 6.403) on the perceived stress scale. Furthermore, it was found that 12.9% of the variance in perceived stress can be explained by the participant’s gender. These findings suggest that gender is an important factor to consider when assessing perceived stress, with female gender being associated with higher levels of perceived stress compared to male gender (see Table 5).

To add to this, the model was found to be statistically significant (F(1,45) = 4.460, *p* = 0.040), suggesting that the number of children may be a predictor of marital cohesion as perceived by parents. The coefficient associated with the number of children indicated a positive relationship between these variables, meaning that, on average, an increase in the number of children was associated with an increase in perceived marital cohesion. However, it is important to note that only 7% (Table 6) of the variance in marital cohesion can be explained by the number of children. On the other hand, no statistically significant values were observed between the variables, indicating that the number of children did not represent a significant predictor of flexibility in this context (see Table 6).

Finally, the results indicate that the model was not statistically significant, suggesting that the diagnosis type may not be a significant predictor of perceived stress. While 62.7% of the variance in perceived stress is explained by the diagnosis type, the model itself was not significant, indicating that other factors not included in the analysis may have a greater influence on perceived stress than the diagnosis type.

## 4. Discussion

First, the hypothesis that the presence of children with SENs would be associated with lower marital cohesion was not confirmed, as no statistically significant correlations were found between the variables. These results align with other studies that also found no significant differences or associations between these two groups [11,36]. However, studies by Martin and Cole [37], Marciano et al. [38], and Hartley et al. [39] report different findings, with both positive and negative correlations between SENs diagnosis and marital cohesion. In the current study, the most frequently observed type of cohesion in both groups was “Separated,” while the least common were “Enmeshed” in the SENs group and “Disengaged” in the other group. The absence of significant correlations suggests that marital cohesion may be influenced by factors beyond the diagnosis itself, such as family dynamics and coping strategies. This emphasizes the importance of therapeutic approaches that promote the development of healthy and resilient relationships while considering individual characteristics.

Regarding flexibility, the results did not support the hypothesis that parents of children with SENs exhibit greater flexibility. Previous studies show divergent results on this topic, with some indicating higher flexibility levels among parents of children with SENs [39], while others demonstrate no significant differences between the groups [36]. The absence of a correlation with the SENs diagnosis may suggest that factors such as social support, emotional resources, and socioeconomic status may have a greater influence on flexibility. The most observed type of flexibility in the SENs group was “Flexible,” while the least observed was “Structured.” In contrast, the second group showed a higher frequency of “Structured” and a lower frequency of “Rigid.” These findings highlight the importance of clinical interventions that address the general challenges of parenting, promoting resilience and adaptive coping strategies for all parents, regardless of whether they have children with SENs.

The third hypothesis, which anticipated higher perceived stress among parents of children with SENs, was also not confirmed, contradicting previous studies [14,40]. These results may be attributed to the fact that both groups reported high levels of perceived stress, and the fact that other relevant variables, such as gender and age, were not considered in the analysis. According to the qualitative description of the scale, the SENs group showed a higher incidence of “High Stress,” while the second group reported “Moderate Stress.” This underscores the need for personalized clinical interventions that consider variables such as age, gender, and individual coping mechanisms, and which aim to promote the emotional well-being of parents and the proper care of children.

The fourth hypothesis proposed that perceived stress mediates the relationship between the child’s diagnosis (concerning special education needs) and the parents’ cohesion and flexibility, but it was not supported. While the perceived stress was found to be a significant predictor of lower marital cohesion (b = −0.6506, *p* < 0.001) and lower marital flexibility (b = −0.4144, *p* = 0.0056), no significant mediation was observed in the relationships between having children with SENs and the marital cohesion or flexibility of the parents. These findings did not support the previous literature regarding the mediation in this field [7,41,42].

The lack of direct and indirect associations between the SENs diagnosis and these variables, including perceived stress, highlights the importance of considering additional factors in the analysis of these factors. Specifically, social support and coping strategies are likely to play a crucial role in how parents adapt to the challenges associated with having children with SENs. Higher levels of social support have been shown to mitigate the impact of stress, potentially buffering its negative effects on marital dynamics [3]. Moreover, the resilience capacity within these families—fostered by effective coping mechanisms—may be a key factor in promoting marital cohesion and flexibility [43]. Adopting a more comprehensive approach is essential to providing tailored support to families with children diagnosed with SENs [3,7].

Analysis of the sociodemographic variables revealed a relationship between gender and the levels of perceived stress, with females reporting higher values. These findings align with previous studies supporting that mothers have higher levels of stress and of perceived stress regarding disabled children [17,44,45]. The number of children also appeared to be associated with greater marital cohesion [5]. However, it is important to interpret this positive relationship cautiously, given the small variance that was explained (7%). This suggests that, while there is an association, other factors not captured by this analysis may have a more significant impact on marital cohesion. Regarding the type of diagnosis, no significant associations were found with the parents’ perceived stress, which contradicts earlier research [46,47,48,49]. The observed patterns emphasize the significance of incorporating sociodemographic factors into clinical interventions. Customizing support to address individual characteristics, such as gender and family size, can improve marital cohesion and reduce the stress in families with children diagnosed with SENs.

### Limitations of This Study

Finally, it is fundamental to contextualize the limitations of the present study. First, its cross-sectional nature prevents the establishment of causal relationships between the variables. A longitudinal design would enable the examination of changes in perceived stress, marital cohesion, and flexibility over time, offering a more comprehensive understanding of the dynamic interplay among these variables in families raising children with SENs. Future research should adopt longitudinal approaches to investigate fluctuations in stress levels and their potential long-term impact on marital functioning. The sample cannot be considered representative, as it was selected through convenience sampling, and the number of participants was small and disproportionate. Additionally, the online data collection method also restricted the opportunity to clarify any doubts, which may have influenced the responses. The online data collection represents an additional limitation of this study, as it compromised the clarification of doubts while participants complete the questionnaire and restricted the participation of individuals without internet access. The Perceived Stress Scale only measures stress levels from the past month, meaning it does not capture potential variations in stress over time or account for events that could have influenced the results during the data collection period. Furthermore, the limited number of studies on this subject, especially in Portugal, restricts the ability to compare results. Future avenues of research may also include machine learning analysis to use algorithms to check for potential variation, as mentioned above, in order to predict and avoid behaviors.

This study aims to analyze the impact of special educational needs (SEN) in a comprehensive manner, including various diagnoses such as cerebral palsy, dysorthography, and low vision disabilities, without focusing exclusively on autism spectrum disorder, as is the case in most existing studies. Furthermore, a mediation model was developed to analyze whether perceived stress acts as a mediator in the relationship between SENs diagnosis, marital cohesion, and parental adaptability. This model, for the Portuguese context, may provide new insights into family and marital dynamics, considering that it has not been observed in previous studies. Lastly, this study also holds significant practical relevance, as it highlights the importance of prioritizing the well-being of couples who are raising children with SENs. In this regard, support programs should consider interventions that strengthen the marital relationship of couples, as this can enhance childcare. Implementing peer support groups, where parents can share experiences and strategies, may reduce their emotional stress and decrease social isolation. Additionally, training programs on stress management, coping strategies, and conflict resolution can help increase family resilience and promote greater marital cohesion. Finally, collaboration with healthcare professionals and local organizations is essential to providing emotional support and offering opportunities for parental self-care.

## Figures and Tables

**Table 1:** Spearman’s correlation coefficient between SENs, real cohesion, ideal cohesion, and ideal flexibility.

Variables	1	2	3	4	5	6	7	8	9	10	11	12	13	14	15	16	17
**1. SEN**	-	−0.154	−0.095	−0.161	−0.237	−0.120	−0.011	0.065	−0.188	−0.161	−0.075	−0.039	−0.164	−0.150	−0.057	−0.050	−0.160
**2. COHE.R**	-	-	0.857 **	0.571 **	0.824 **	0.731 **	0.842 **	0.278 **	0.517 **	0.571 **	0.435 **	0.593 **	0.623 **	0.352 **	0.473 **	0.284 *	0.207
**3. BOND.R**	-	-	-	0.536 **	0.674 **	0.447 **	0.636 **	0.359 **	0.472 **	0.536 **	0.395 **	0.337 *	0.502 **	0.277 *	0.275 *	0.284 *	0.208
**4. SUPP.R**	-	-	-	-	0.447 **	0.314 *	0.334 *	0.172	0.436 **	1.000 **	0.220	0.276 *	0.393 **	0.344 **	0.399 **	0.356 **	0.223
**5. BOUN.R**	-	-	-	-	-	0.644 **	0.634 **	0.239	0.442 **	0.447 **	0.341 **	0.435 **	0.530 **	0.324 *	0.360 **	0.125	0.275 *
**6. TIME.R**	-	-	-	-	-	-	0.587 **	0.255 *	0.474 **	0.314 *	0.449 **	0.744 **	0.517 **	0.417 **	0.550 **	0.324 *	0.250 *
**7. INT.R**	-	-	-	-	-	-	-	0.165	0.297 *	0.334 *	0.279 *	0.470 **	0.529 **	0.201	0.347 **	0.122	0.091
**8. COHE.I**	-	-	-	-	-	-	-	-	0.154	0.172	0.241	0.191	0.310 *	0.133	−0.013	0.085	0.226
**9. BOND.I**	-	-	-	-	-	-	-	-	-	0.436 **	0.838* *	0.620 **	0.770 **	0.702 **	0.665 **	0.648 **	0.556 **
**10. SUPP.I**	-	-	-	-	-	-	-	-	-	-	0.220	0.276 *	0.393 **	0.344 **	0.399 **	0.356 **	0.223
**11. BOUN.I**	-	-	-	-	-	-	-	-	-	-	-	0.621 **	0.767 **	0.525 **	0.510 **	0.494 **	0.415 **
**12. TIME.I**	-	-	-	-	-	-	-	-	-	-	-	-	0.623 **	0.506 **	0.677 **	0.550 **	0.276 *
**13. INT.I**	-	-	-	-	-	-	-	-	-	-	-	-	-	0.680 **	0.671 **	0.476 **	0.563 **
**14. FLEX.I**	-	-	-	-	-	-	-	-	-	-	-	-	-	-	0.855 **	0.624 **	0.909 **
**15. LEAD.I**	-	-	-	-	-	-	-	-	-	-	-	-	-	-	-	0.615 **	0.594 **
**16. NEG.I**	-	-	-	-	-	-	-	-	-	-	-	-	-	-	-	-	0.398 **
**17. ROLES.I**	-	-	-	-	-	-	-	-	-	-	-	-	-	-	-	-	-

**Note**: COHE.R = real cohesion; BOND.R = real emotional bond; SUPP.R = real support; BOUN.R = real family boundaries; TIME.R = real free time and friends; INT.R = real interests and recreational activities; COHE.I = ideal cohesion; BOND.I = ideal emotional bond; SUPP.I = ideal support; BOUN.I = ideal family boundaries; TIME.I = ideal free time and friends; INT.I = ideal interests and recreational activities; FLEX.I = ideal flexibility; LEAD.I = ideal leadership and control; NEG.I = ideal negotiation; ROLES.I = ideal roles and rules. * *p* < 0.05; ** *p* < 0.01.

**Table 2:** Pearson correlation coefficient between SENs, real flexibility, and perceived stress.

Variables	1	2	3	4	5	6
**1. SEN**	-	−0.161	−0.220	−0.136	−0.057	0.77
**2. FLEX.R**	-	-	0.861 **	0.791 **	0.865 **	−0.408 *
**3. LEAD.R**	-	-	-	0.613 **	0.518 **	−0.456 **
**4. NEG.R**	-	-	-	-	0.607 **	−0.374 *
**5. ROLES.R**	-	-	-	-	-	−0.231
**6. PSS**	-	-	-	-	-	-

**Note**: FLEX.R = real flexibility; LEAD.R = real leadership and control; NEG.R = real negotiation; ROLES.R = real roles and rules; PSS = perceived stress scale. * *p* ≤ 0.05; ** *p* < 0.01.

**Table 3:** Mediation between perceived stress, SENs, and cohesion.

	B	SD	t	*p*	LLCI	ULCI
**Path a:** SENs > Perceived Stress	1.0870	2.0996	0.5177	0.6072	−3.1419	5.3158
**Path b:** Perceived Stress > Cohesion	−0.6506	0.1221	−5.3304	0.0000	−0.8966	−0.4046
**Path c—Total Effect:** SENs > Cohesion	−1.6920	2.1807	−0.7759	0.4419	−6.0842	2.7002
**Path c’– Direct Effect:** SENs > Cohesion	−0.9849	1.7242	−0.5712	0.5708	−4.4597	2.4900
**Indirect Effect (5000 Bootstrap)**	−0.7072	1.4606			−3.7310	1.9633

**Note**: *LLCI* = Lower limit of the 95% confidence interval; *ULCI* = upper limit of the 95% confidence interval. The coefficients and effects presented are not standardized.

**Table 4:** Mediation between perceived stress, SENs, and flexibility.

	B	SD	t	*p*	LLCI	ULCI
**Path a:** SENs > Perceived Stress	1.0870	2.0996	0.5177	0.6072	−3.1419	5.3158
**Path b:** Perceived Stress > Flexibility	−0.4144	0.1423	−2.9121	0.0056	−0.7012	−0.1276
**Path c—Total Effect:** SENs > Flexibility	−2.3659	2.1643	−1.0931	0.2801	−6.7252	1.9933
**Path c’–Direct Effect:** SENs > Flexibility	−1.9155	2.0101	−0.9529	0.3458	−5.9667	2.1357
**Indirect Effect (5000 Bootstrap)**	−0.4504	0.9605			−2.5786	1.3209

**Note**: *LLCI* = Lower limit of the 95% confidence interval; *ULCI* = upper limit of the 95% confidence interval. The coefficients and effects presented are not standardized.

**Table 5:** Simple linear regression between gender and perceived stress.

	β	R^2^ Adjusted	SD	t	*p*
**Gender**	−5.734	0.129	2.051	−2.796	0.008

**Table 6:** Simple linear regression between number of children and cohesion.

	β	R^2^ Adjusted	SD	t	*p*
**Nº children**	3.133	0.070	1.482	2.112	0.040

## Data Availability

Data generated and analyzed during this study are included and clearly identified in this article.
